# High-Sensitivity Cardiac Troponin I Following Gender-Affirming Hormone Therapy in Transgender Adults

**DOI:** 10.1001/jamanetworkopen.2025.37205

**Published:** 2025-10-13

**Authors:** Ada S. Cheung, Ingrid Bretherton, Shalem Y. Leemaqz, Sathya Perera Wedithanthrige, Leah Singh, Cherie Chiang

**Affiliations:** 1 Department of Medicine, The University of Melbourne, Heidelberg, Victoria, Australia; 2 Department of Endocrinology, Austin Health, Heidelberg, Victoria, Australia; 3 Flinders University, South Australia, Australia; 4 Department of Pathology, Royal Melbourne Hospital, Parkville, Victoria, Australia

## Abstract

This case-control study examines high-sensitivity cardiac troponin I levels in transgender men and women over 12 months of gender affirming hormone theraoy compared with cisgender men and women

## Introduction

High-sensitivity cardiac troponin I (hs-cTnI) predicts cardiovascular events, such as myocardial infarction, heart failure, and cardiovascular death, even when values are within reference limits.^1^ Guidelines recommend sex-specific reference ranges due to higher troponin levels in men, reflecting greater cardiac muscle mass.^2^ However, it remains unclear which reference range is appropriate for transgender people using gender-affirming hormone therapy (GAHT).

We hypothesized that since estradiol and testosterone are not thought to directly affect cardiac mass, hs-cTnI levels would remain stable in transgender individuals starting GAHT. We aimed to prospectively evaluate hs-cTnI levels in transgender men and women over 12 months of GAHT compared with cisgender men and women.

## Methods

This case-control study followed the STROBE reporting guideline. We conducted a 12-month prospective parallel cohort study at a tertiary referral hospital. Transgender adults initiating standard-dose GAHT were recruited between May 2017 and July 2022 from outpatient clinics specializing in transgender health in Australia. Cisgender adults were recruited via community advertising. Exclusion criteria included pregnancy, thromboembolic disease, liver disease, conditions affecting bone health (as recruitment was primarily for a bone health study), or use of medications influencing bone metabolism or body composition (eg, glucocorticoids) during the study. All participants provided written informed consent. The study was approved by the Austin Health Human Research Ethics Committee.

Fasting morning blood samples were collected at baseline and 12 months, frozen, and batch-analyzed using the ARCHITECT i2000 hs-cTnI assay (Abbott Diagnostics ). Reference ranges were less than 26 ng/L for males and less than 16 ng/L for females (to convert to grams per liter, multiply by 1). Testosterone and estradiol were measured using electrochemiluminescence immunoassays.

Statistical analyses included linear mixed-effects models adjusted for age, with random intercepts to account for repeated measures. Hs-cTnI was log-transformed to approximate normality and presented as ratio of geometric means (RGM) with 95% CIs with *P* < .05 considered significant. Data were analyzed from May 2024 to August 2025.

## Results

This study included 152 participants (median [IQR] age, 26 [23-34] years); 103 had paired data at 12 months (Table). In transgender women, estradiol increased from 31 to 102 pg/mL (to convert to picomoles per liter, multiply by 3.671), and testosterone decreased from 453 to 26 ng/dL (to convert to nanomoles per liter, multiply by 0.0347). In transgender men, testosterone rose from 35 to 542 ng/dL, while estradiol dropped from 95 to 38 pg/mL.

**Table.** Participant Characteristics

Variable	Participant, No. (%)		*P* value		No. (%)		*P* value	
	Trans women (n = 28)	Cis women (n = 46)	Baseline	Change over time	Trans men (n = 31)	Cis men (n = 47)	Baseline	Change over time
hs-cTnI, median (IQR), ng/L^a^								
Baseline	1.5 (0.9-2.7)	0.5 (0.1-1.3)	<.001	.21	0.5 (0.2-1.0)	1.2 (0.8-2.5)	<.001	.32
12 mo	0.7 (0.4-1.9)	0.4 (0.1-0.9)			0.8 (0.6-1.3)	1.4 (0.7-2.8)		
Age, median (IQR), y	27.0 (22.8-35.8)	26.5 (24-39)	.98	NA	23.0 (21.5-28.5)	28.0 (23.5-37.5)	.02	NA
Medical history								
Diabetes	0	1 (2)	NA	NA	0	0	NA	NA
Hypertension	4 (14)	1 (2)	.04	NA	1 (3)	1 (2)	.76	NA
Hypercholesterolemia	3 (11)	0	.02	NA	2 (6)	1 (2)	.33	NA
Cardiac disease	0	0	NA	NA	0	0	NA	NA
Stroke or TIA	0	0	NA	NA	0	0	NA	NA
Kidney impairment, eGFR <60 ml/min/1.73m^2^	0	0	NA	NA	0	0	NA	NA

Abbreviations: eGFR, estimated glomerular filtration rate; hs-cTnI, high–sensitivity cardiac troponin I; NA, not applicable; TIA, transient ischemic attack.

SI conversion factors: To convert estradial to picomoles per liter, multiply by 3.671; hs-cTnI to grams per liter, multiply by 1; testosterone to nanomoles per liter, multiply by 0.0347.

^a^ Baseline hs-cTnI levels did not differ significantly between transgender women and cisgender men, or between transgender men and cisgender women.

Hs-cTnI decreased in transgender women over 12 months and was not statistically different from cisgender women (RGM, 2.24 [95% CI, 0.63-7.92]; *P* = .21), but was significantly 78% lower than cisgender men (RGM, 0.22 [95% CI, 0.07-0.70]; *P* = .01) (Figure). In transgender men, hs-cTnI increased and was like cisgender men (RGM, 0.56 [95% CI, 0.18-1.77]; *P* = .32), but significantly higher than cisgender women (RGM, 7.39 [95% CI, 2.05-26.63]; *P* = .003) (Figure).

**Figure.**
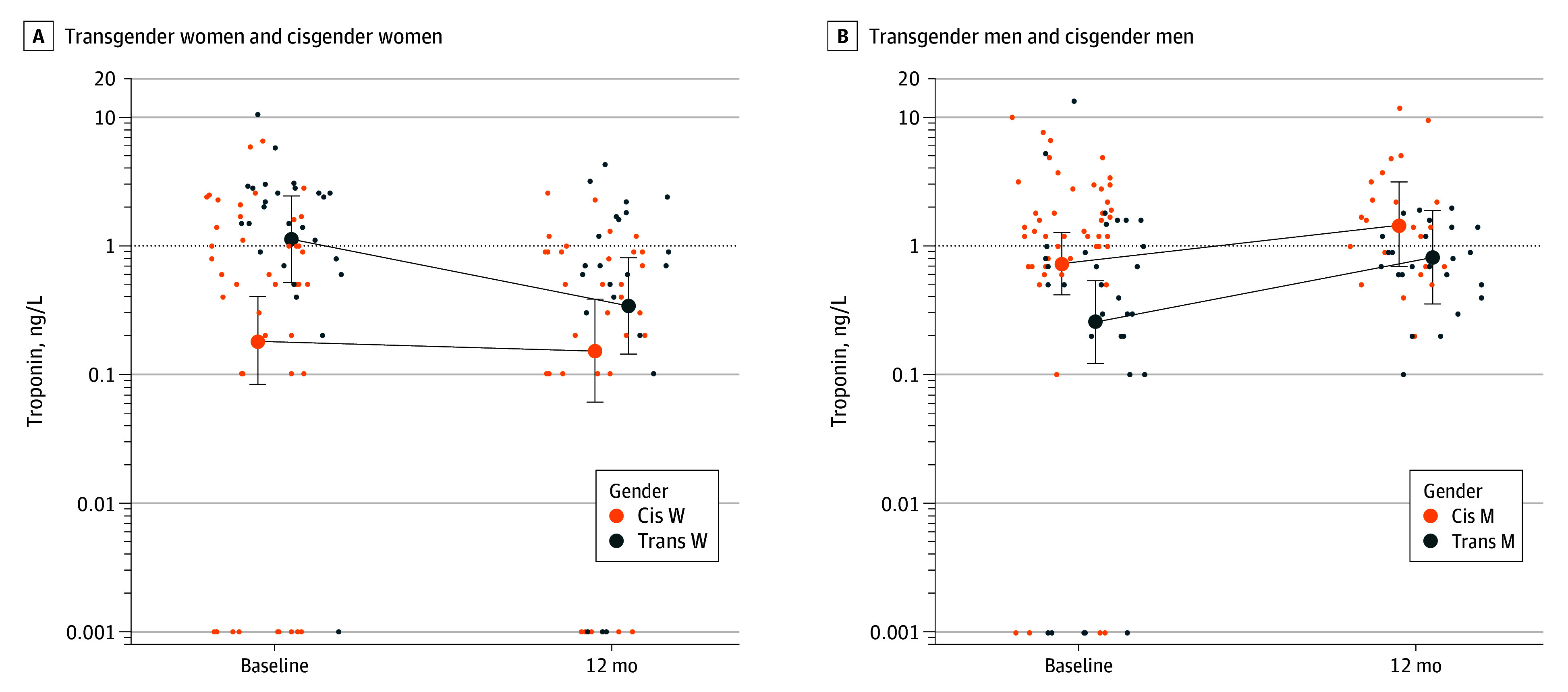
High-Sensitivity Cardiac Troponin I Concentrations at Baseline and 12 Months After Starting Gender-Affirming Hormone Therapy for Transgender Women (Trans W) and Cisgender Women (Cis W) and Transgender Men (Trans M) and Cisgender Men (Cis M) To convert troponin to nanogram per milliliter, multiply by 0.001. The dots represent the estimated marginal means, and the whiskers indicate the 95% CI.

## Discussion

Our findings suggest that hs-cTnI levels shift toward the affirmed gender after 12 months of GAHT, suggesting potential effects of GAHT on cardiac mass or function. Prior echocardiographic studies found transgender women have smaller ventricular diameters than cisgender men,^3^ and higher testosterone is associated with greater left ventricular mass.^4^

Our findings align with earlier cross-sectional research showing higher hs-cTnI in transgender men than transgender women, although earlier studies lacked baseline data or cisgender comparators.^5^ All participants were healthy, yet post-GAHT hs-cTnI aligned with affirmed gender. Importantly, transgender patients presenting with acute myocardial infarction have exceeded both male and female cutoffs,^6^ suggesting either threshold may be safe, but affirmed gender ranges could reduce confusion and distress.

This first longitudinal study with cisgender comparators is limited by only baseline and 12-month sampling (missing earlier or later changes) and no cardiac imaging. Hs-cTnI changes significantly after 12 months of standard GAHT, supporting affirmed gender reference ranges only after this duration. Further research should examine structural or functional changes mechanisms and long-term cardiovascular implications.

## Notes

Supplement.
Data Sharing Statement
