# Supplementary material for: High-Sensitivity Cardiac Troponin I Following Gender-Affirming Hormone Therapy in Transgender Adults
Source: JAMA Netw Open. 2025 Oct 13;8(10):e2537205. doi: 10.1001/jamanetworkopen.2025.37205 (PMC12519299; doi:10.1001/jamanetworkopen.2025.37205)
Supplement: Supplement. — Data Sharing Statement [file jamanetwopen-e2537205-s001.pdf]

Data Sharing Statement

Data
Data available: Yes
Data types: Deidentified participant data
How to access data: Deidentified participant data can be obtained by request to the corresponding author via email to adac@unimelb.edu.au
When available: With publication

Supporting Documents
Document types: None

Additional Information
Who can access the data: Researchers whose proposed use of the data has been approved.
Types of analyses: For purposes to improve the health and wellbeing of the trans community.
Mechanisms of data availability: After approval of a proposal and with a signed data access agreement.
